# Addendum: Precise tuning of gene expression levels in mammalian cells

**DOI:** 10.1038/s41467-019-10615-0

**Published:** 2019-06-10

**Authors:** Yale S. Michaels, Mike B. Barnkob, Hector Barbosa, Toni A. Baeumler, Mary K. Thompson, Violaine Andre, Huw Colin-York, Marco Fritzsche, Uzi Gileadi, Hilary M. Sheppard, David J. H. F. Knapp, Thomas A. Milne, Vincenzo Cerundolo, Tudor A. Fulga

**Affiliations:** 1 Weatherall Institute of Molecular Medicine, Radcliffe Department of Medicine, University of Oxford, Oxford, OX3 9DS UK; 2 MRC Human Immunology Unit, Weatherall Institute of Molecular Medicine University of Oxford, Oxford, OX3 9DS UK; 3 Department of Biochemistry, University of Oxford, Oxford, OX1 3QU UK; 4 Kennedy Institute of Rheumatology, Nuffield Department of Orthopaedics, Rheumatology and Musculoskeletal Sciences, Oxford, OX3 7FY UK; 5 School of Biological Sciences, University of Auckland, Auckland, 1050 New Zealand; 6 Weatherall Institute of Molecular Medicine, MRC Molecular Haematology Unit, NIHR Oxford Biomedical Research Centre Programme, University of Oxford, Oxford, OX3 9DS UK

**Keywords:** High-throughput screening, Synthetic biology, Immunology, CRISPR-Cas9 genome editing, miRNAs

## Abstract

Following re-sequencing of the miSFIT constructs used in the paper, two of the construct variants inserted into the 3’UTR of PD-1, namely ‘12C’ and ‘17A, 18G’, have been found to contain additional insertions not present in the other construct variants. The data points corresponding to these constructs in Figs. 2c, f and Supplementary Fig. 9 are therefore no longer valid. However the overall conclusion that step-wise control over gene expression levels using the miSFIT constructs remains unaffected by these errors. Updated versions of Fig. 2 and Supplementary Fig. 9 are presented in the accompanying Addendum.

Addendum to: *Nature Communications* 10.1038/s41467-019-08777-y, published online 18 February 2019.

Following re-sequencing of the miSFIT constructs used in the paper, two of the construct variants inserted into the 3′UTR of PD-1, namely ‘12C’ and ‘17A, 18G’, have been found to contain additional insertions not present in the other construct variants. The data points corresponding to these constructs in Fig. 2c, f and Supplementary Fig. 9 are therefore no longer valid. However the overall conclusion that the miSFIT system allows step-wise control over gene expression levels remains unaffected by these errors. Updated versions of Fig. 2 and Supplementary Fig. 9 are presented below as Figs. 1 and 2 respectively.

**Figure 1:**
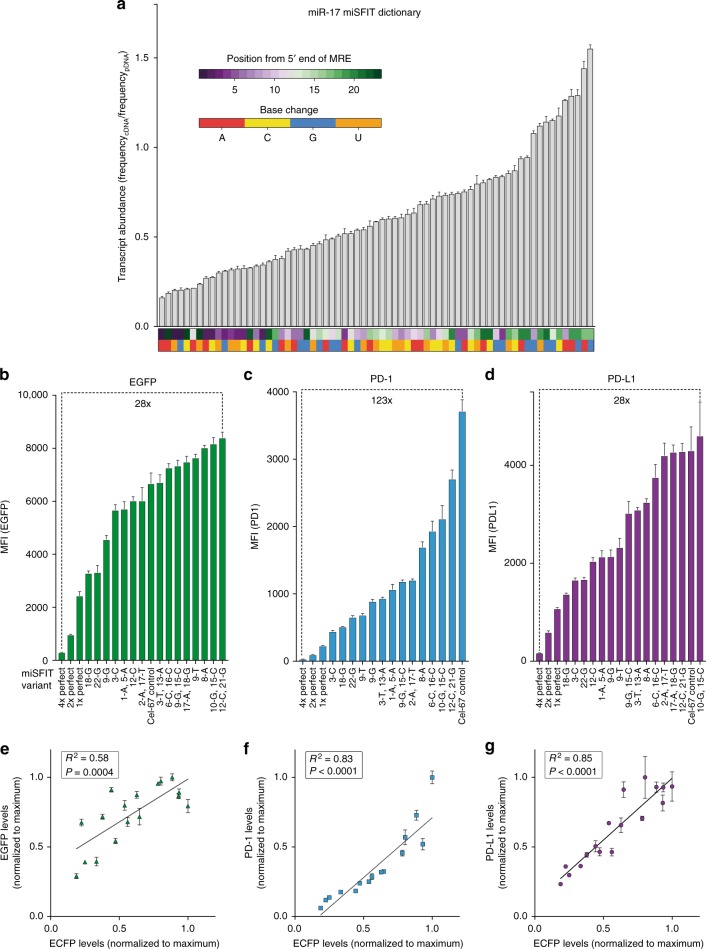
Fig. 1

**Figure 2:**
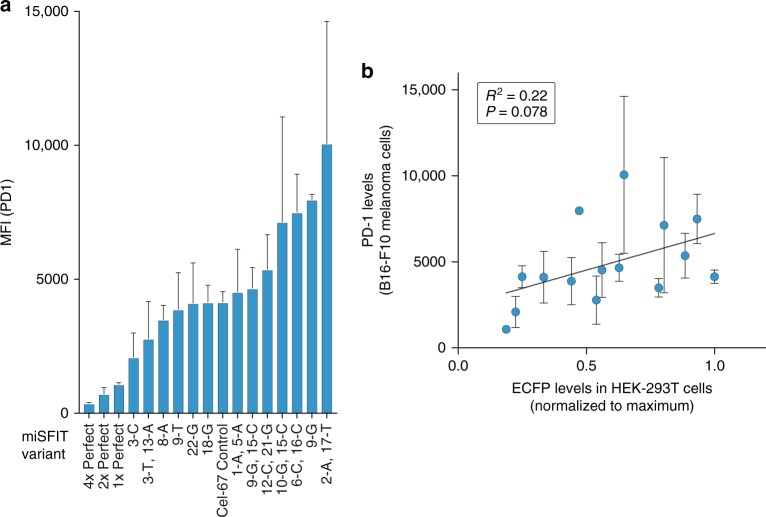
Fig. 2

