# Supplementary material for: Precise tuning of gene expression levels in mammalian cells
Source: Nat Commun. 2019 Feb 18;10:818. doi: 10.1038/s41467-019-08777-y (PMC6379387; doi:10.1038/s41467-019-08777-y)
Supplement: Supplementary file 1 — Supplementary Information [file 41467_2019_8777_MOESM1_ESM.pdf]

Supplementary Information

Michaels et al.
Precise tuning of gene expression levels in mammalian cells

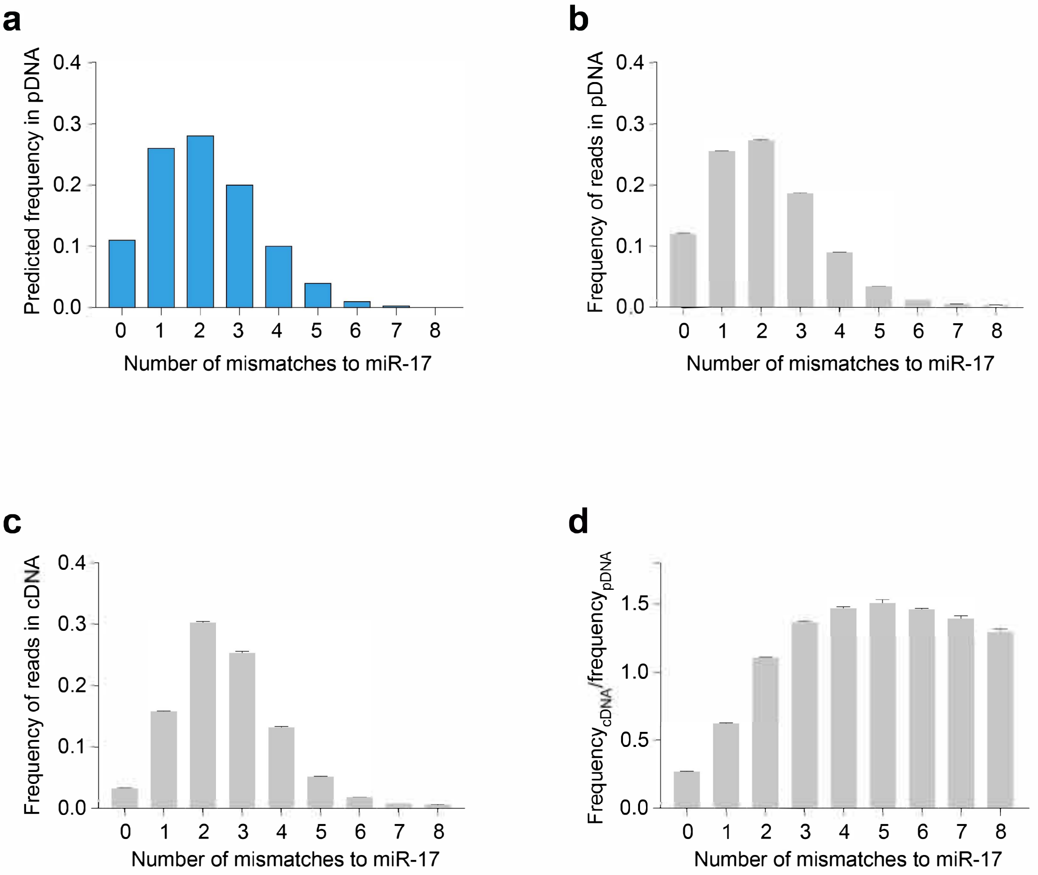

Supplementary Figure 1. High-throughput study of the effect of mismatches on MRE functionality. (a) Expected probability distribution for the number of mismatches per MRE in the miR-17 MRE variant library (see Fig. 1a). **(b)** Frequency of reads containing between 1 and 8 mismatches to miR-17 in plasmid DNA (pDNA) isolated from HEK-293T cells transfected with the miR-17 MRE library (n = 3 biological replicates, mean + s.d.). **(c)** Frequency of reads containing 1 to 8 mismatches to miR-17 in cDNA libraries from the same pools of cells as in (b) (n = 3 biological replicates, mean + s.d.). **(d)** Average enrichment for MREs with varying numbers of mismatches calculated by dividing frequency of reads in cDNA libraries (c) by the frequency of reads in pDNA libraries (b) (n = 3 biological replicates, mean + s.d.). Source data are provided as a Source Data file.

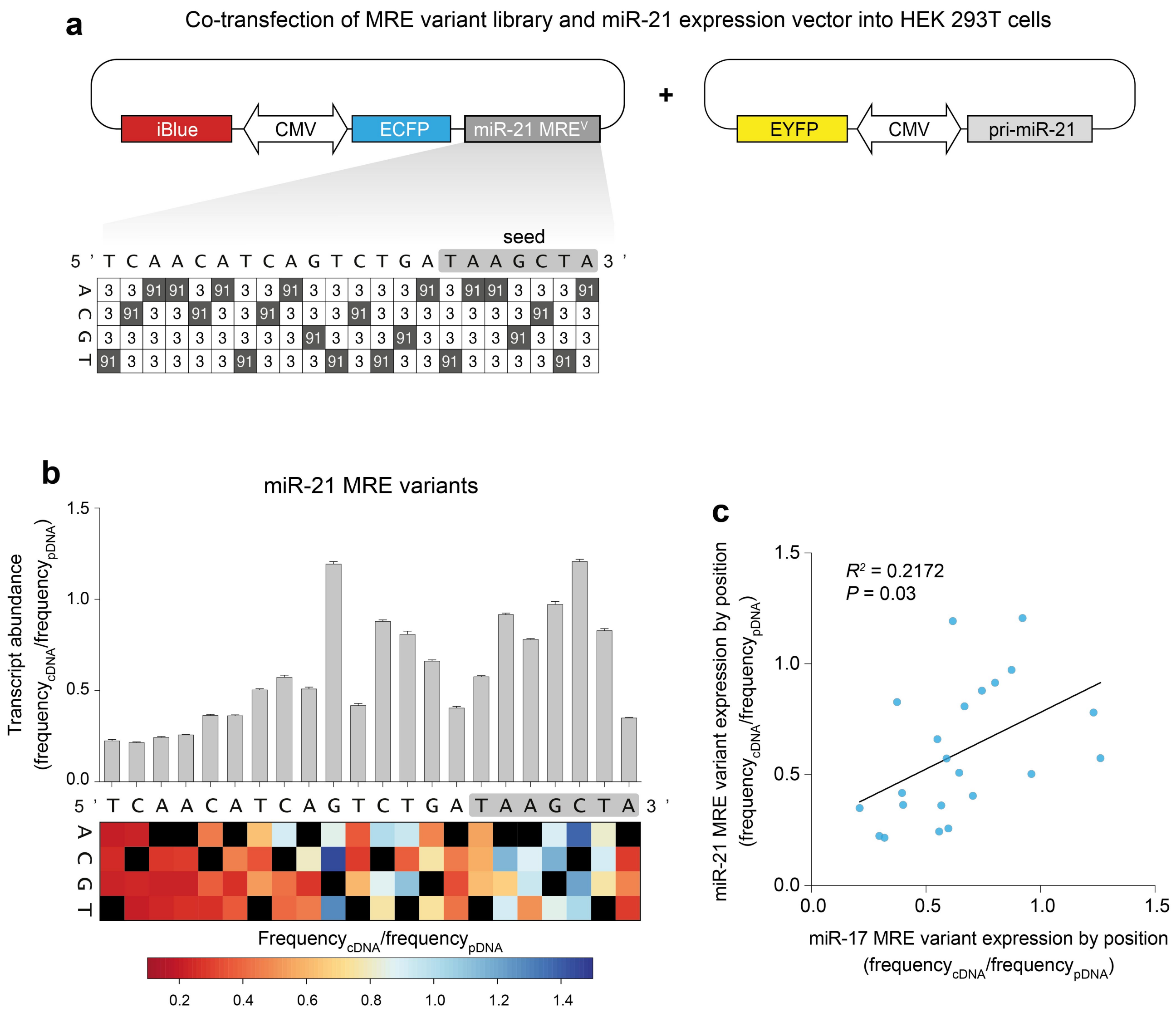

Supplementary Figure 2. Single-nucleotide resolution analysis of the miR-21 MRE regulatory landscape. (a) Schematic representation of the miR-21 MRE library design (compare to miR-17 library in Fig. 1). Values indicate the proportion of nucleotides at each position in the MRE (shaded squares = nucleotides complementary to miR-21). Since miR-21 is expressed at low levels in HEK-293T cells, we overexpressed the primary miR-21 transcript using a plasmid vector which also delivers EYFP as a transfection control. **(b)** Impact of MRE variants on transcript abundance. Bar graph shows the impact of single nucleotide mismatches at each position in the MRE on the strength of reporter silencing by high-throughput sequencing (n = 3 biological replicates, mean + s.d.). Heat-map displays the effect of each possible mismatch by position and reflects the average of three replicates. (black squares = complementary bases) **(c)** Linear regression comparing the impact of mismatches at each position between the miR-17 and miR-21 synthetic MRE variants (n = 22 positions). Source data are provided as a Source Data file.

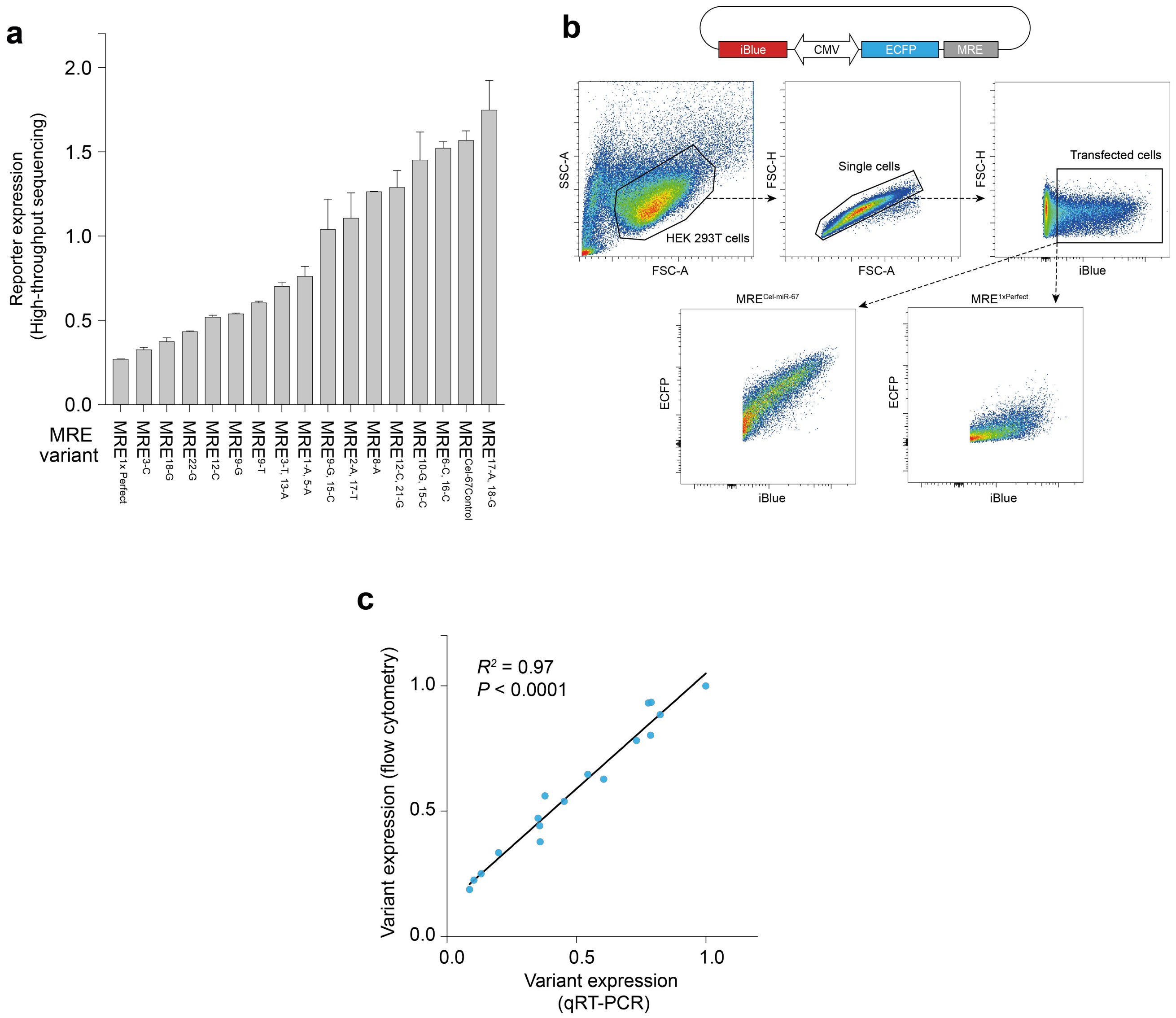

Supplementary Figure 3. RT-qPCR and flow cytometry validate high-throughput MRE screen data. (a) Ranked impact of candidate MRE variants on transcript abundance. To select an unbiased set of miR-17 MREs from our variant library for validation using conventional methods, we transformed the plasmid library (see Fig. 1) into *E. coli* and randomly picked and screened colonies to identify 15 unique single or double variant MREs. Bar graph represents the effect on reporter expression of these variants relative to a perfect miR-17 MRE ($MRE^{1xPerfect}$) and $MRE^{Cel\text{-}67\ Control}$, as determined by the high-throughput sequencing experiment (n = 3 biological replicates, mean + s.d.). **(b)** Flow cytometry validation gating strategy used to generate Supplementary Figure 3c and Fig. 1i. **(c)** Linear regression comparing expression as measured by RT-qPCR to flow cytometry in HEK-293T cells transfected with each of the 15 MRE variants in the validation set (n = 17 variants, $P < 0.0001$, slope differs from 0). Transcript expression was calculated by the ΔΔCT method using iBlue as a reference gene. Flow cytometry expression was calculated by normalizing ECFP expression to iBlue on a single cell basis and taking the mean of that value for each MRE variant. Source data are provided as a Source Data file.

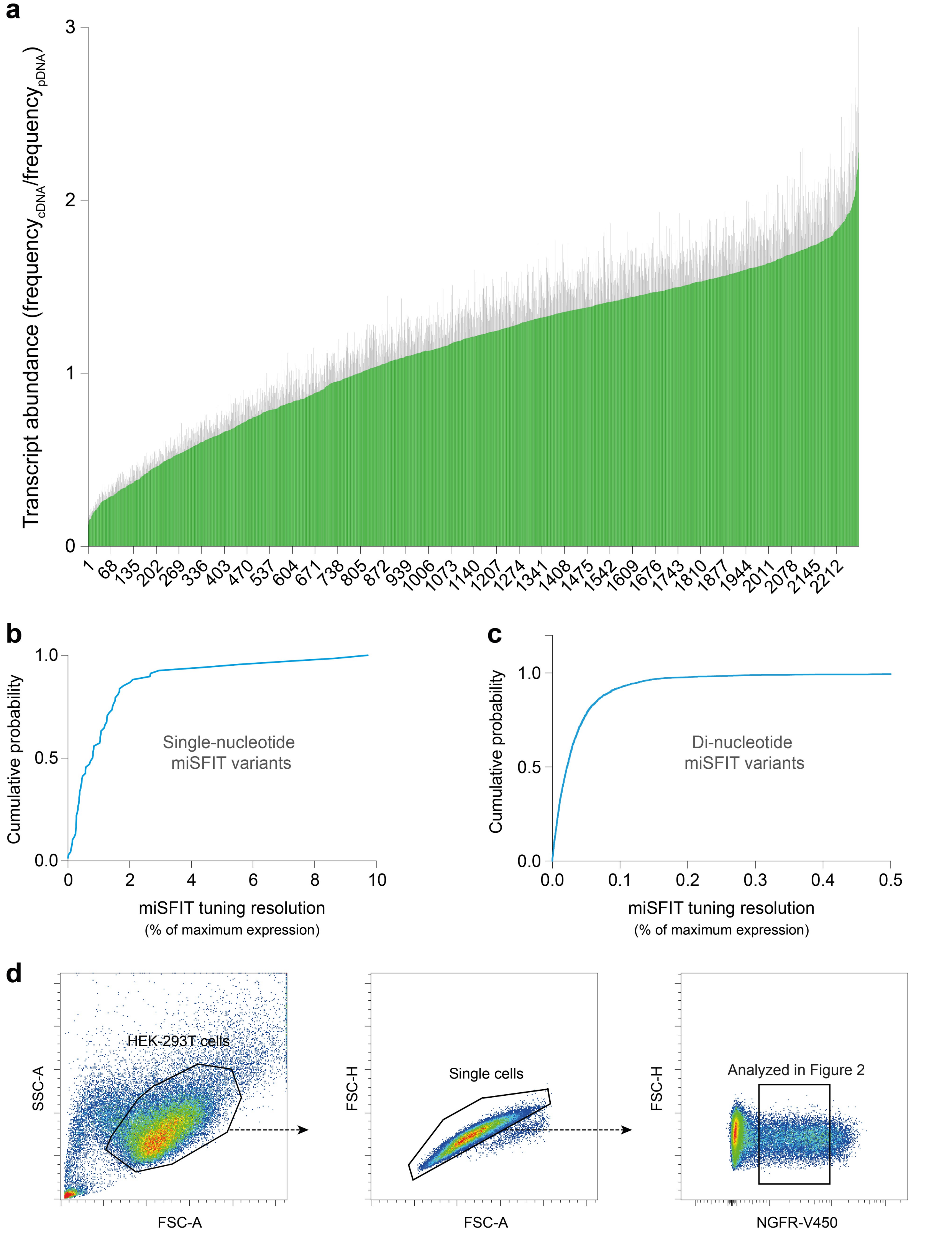

Supplementary Figure 4. Di-nucleotide miSFIT variants provide high-resolution fine-tuning of gene expression. (a) Impact on transcript abundance of all possible di-nucleotide miR-17 miSFIT variants ranked by expression output (n = 3 biological replicates, mean + s.d.). **(b, c)** Cumulative probability distribution of the precision that can be achieved using miSFIT technology. The distribution reflects the difference in ECFP expression between nearest single-nucleotide (b) or di-nucleotide (c) miR-17-MRE variants in HEK-293T cells. **(d)** Flow cytometry gating strategy used to generate the plots in Figure 2b-d. Source data are provided as a Source Data file.

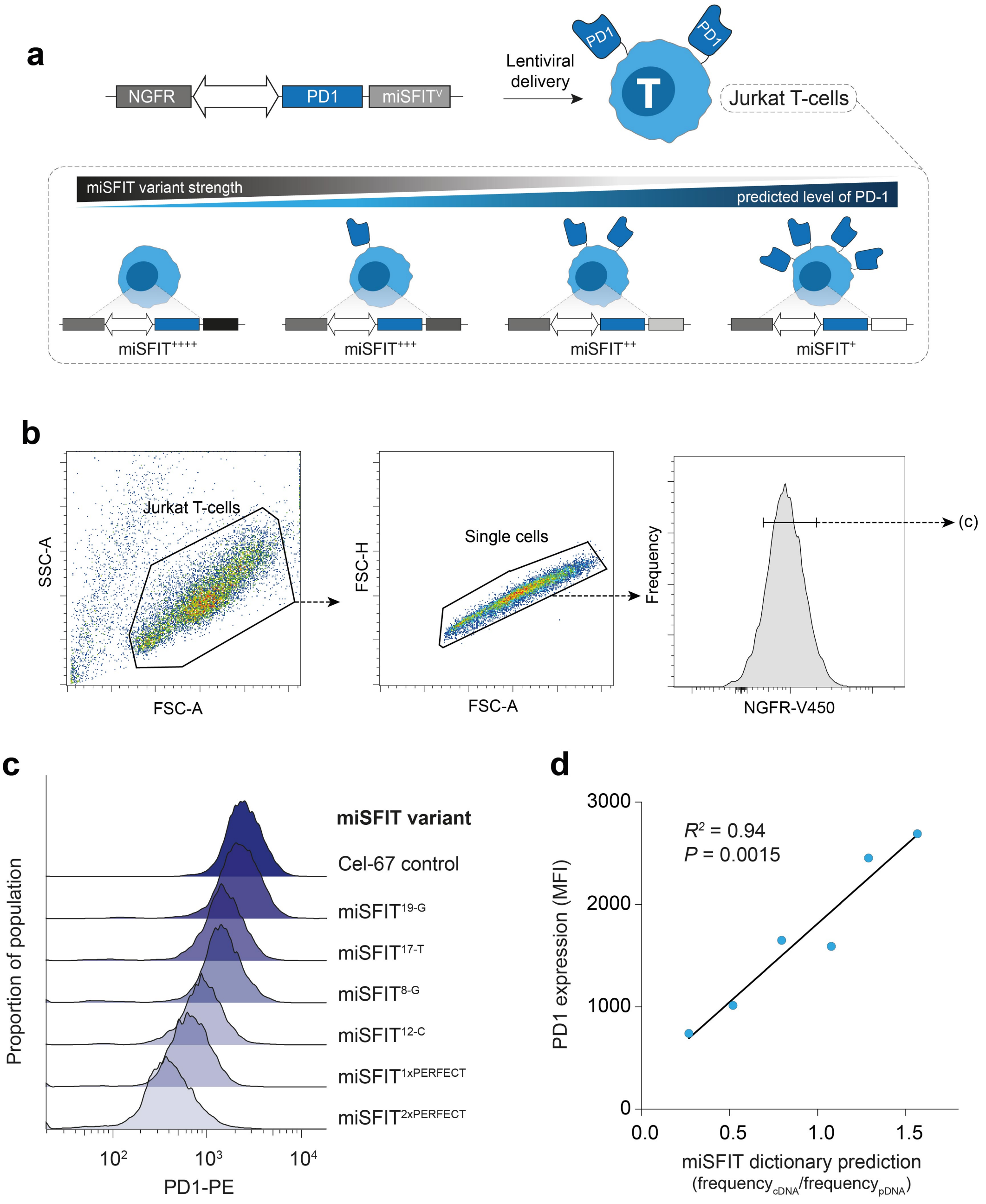

Supplementary Figure 5. Stable tuning of PD-1 expression in Jurkat T-cells. (a) Schematic of miSFIT tuning strategy. PD-1 expression is controlled by various miSFIT variants while NGFR serves as an un-silenced internal control. **(b)** Flow-cytometry gating strategy used to generate the data in (c). **(c)** Flow-cytometry histograms of PD-1 expression on Jurkat T-cell lines transduced with one of six different miSFIT variants (x-axis = $\log_{10}$ transformed PD1-PE fluorescence). **(d)** Correlation between predicted expression in the miSFIT dictionary and observed PD1 expression on Jurkat T-cell lines (n = 6 variants, linear regression). Source data are provided as a Source Data file.

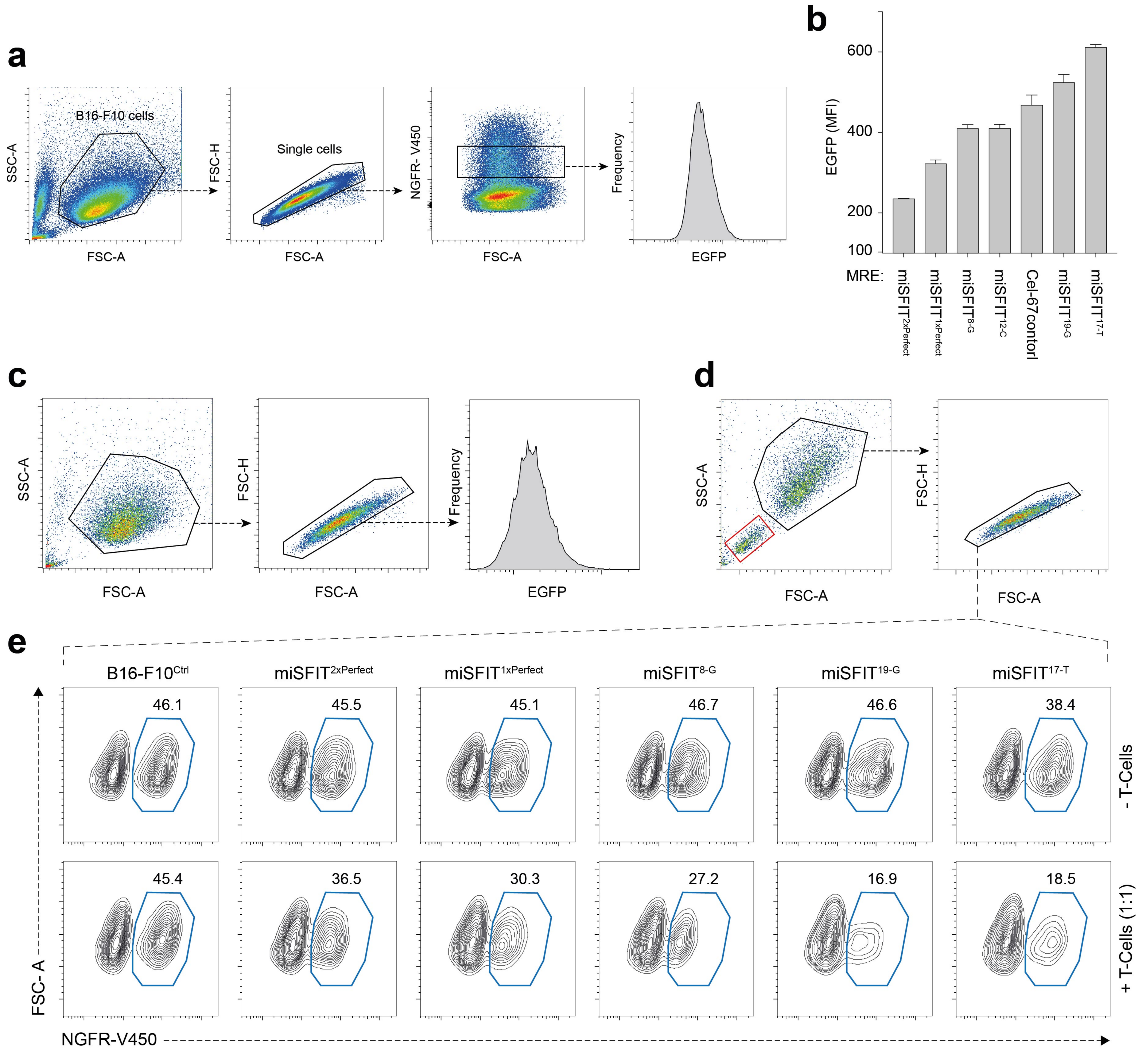

Supplementary Figure 6. Flow cytometry analysis of OVA-T2A-GFP miSFIT cell lines. (a) Flow-cytometry gating strategy used to assess OVA-T2A-EGFP expression following transient transfection of seven different miSFIT variant constructs in B16-F10 cells. This gating strategy corresponds to (b) **(b)** EGFP expression of seven OVA-miSFIT constructs (n = 3 biological replicates, mean +/- s.d.). **(c)** Flow cytometry gating strategy used to analyse miSFIT variant cell lines following lentiviral transduction and cell sorting as shown in Figure 3b. **(d)** Flow cytometry gating strategy used for B16-F10 mixed co-culture experiments shown in Supplementary Figure 6e and Fig 3e (red gate = T-cells). **(e)** NGFR expression following overnight mixed co-cultures of B16-F10 cell lines expressing each of five OVA-miSFIT variants as well as an OVA-negative control cell line with $CD8^+$ OT-I T-cells at 1:1 T-cell: melanoma cell ratio (blue polygon gate = the percentage of $NGFR^+$ (OVA-miSFIT) cells surviving after overnight selection; compare to a 3:1 ratio in Fig. 3e; the results of both experiments are summarized in Fig. 3f). Source data are provided as a Source Data file.

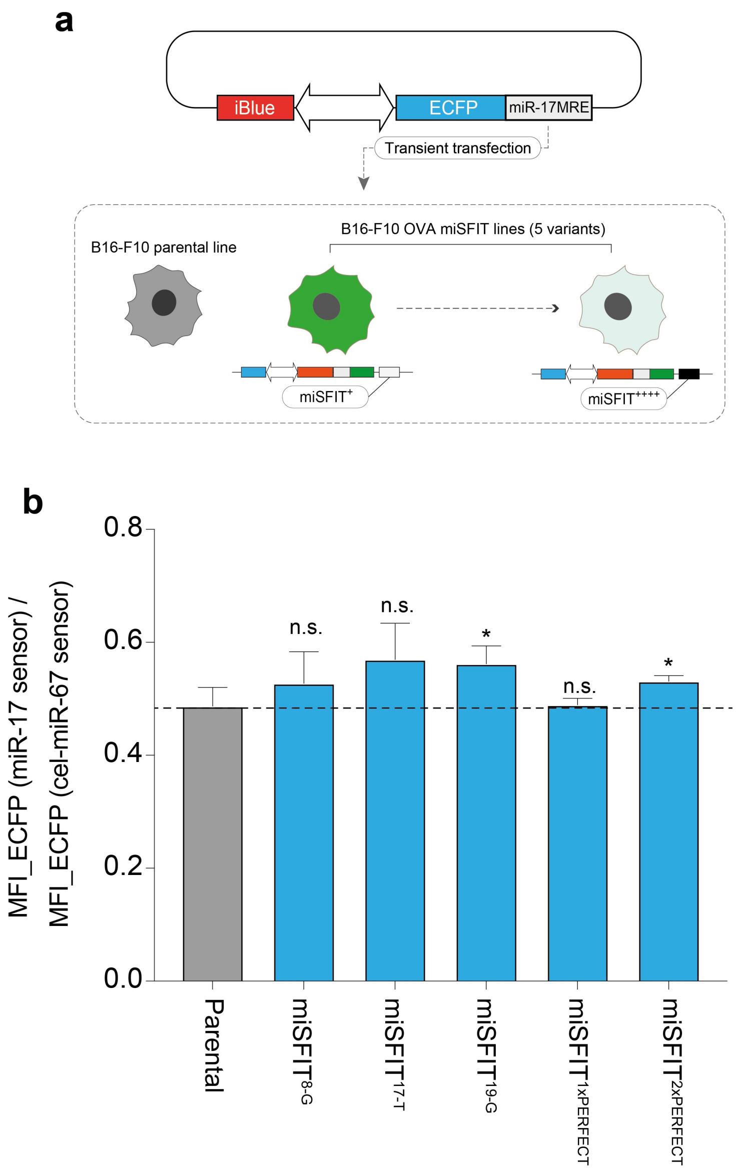

Supplementary Figure 7. miR-17 miSFITs do not act as competitive miRNA sponges. (a) Schematic of miR-17 reporter experiments in B16-F10 OVA-miSFIT cell lines. **(b)** Flow cytometry analysis of miR-17 ECFP reporter expression in B16-F10 cells expressing various OVA-miSFIT variants (n = 3 biological replicates, mean + s.d., *Dunnet's* multiple comparison test vs parental cells, * = $P < 0.05$). Analysed events were gated on iBlue, an internal transfection control. Cell lines are ordered left to right from weakest to strongest miSFIT. Source data are provided as a Source Data file.

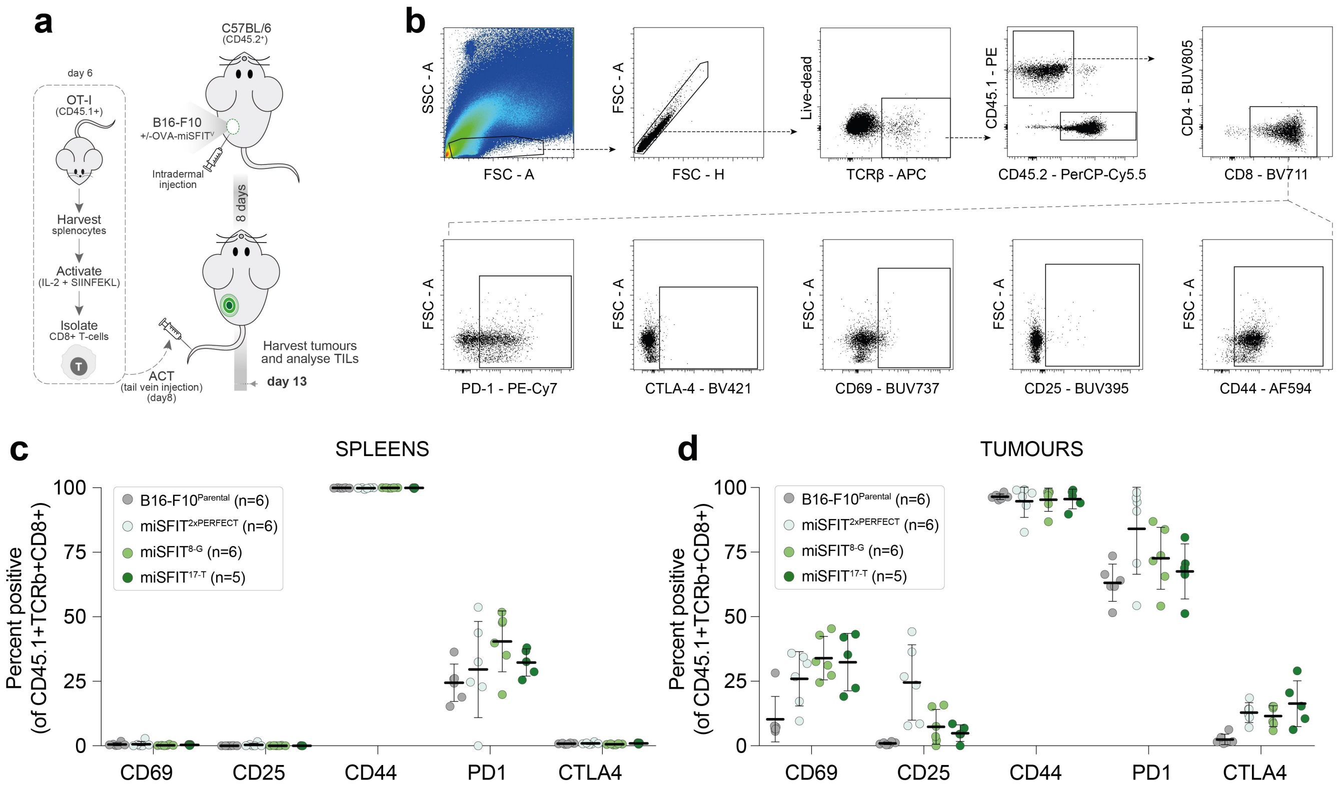

Supplementary Figure 8. Analysis of Tumour Infiltrating Lymphocytes (TILs). (a) Schematic representation of the experimental design employed to assess the effect of OVA expression on Tumour Infiltrating Lymphocytes (TILs). **(b)** Flow cytometry gating strategy used to analyse TILs and produce data shown in Supplementary Figure 8c,d and Fig. 4d. Gates were drawn based on fluorescence-minus-one controls. OT-I donor mice are CD45.1^{+} while recipient mice are CD45.2^{+}. **(c, d)** Cell surface marker expression on T-cells harvested from recipient mouse spleens **(c)** and tumours **(d)** (mean +/- s.d.). Source data are provided as a Source Data file.

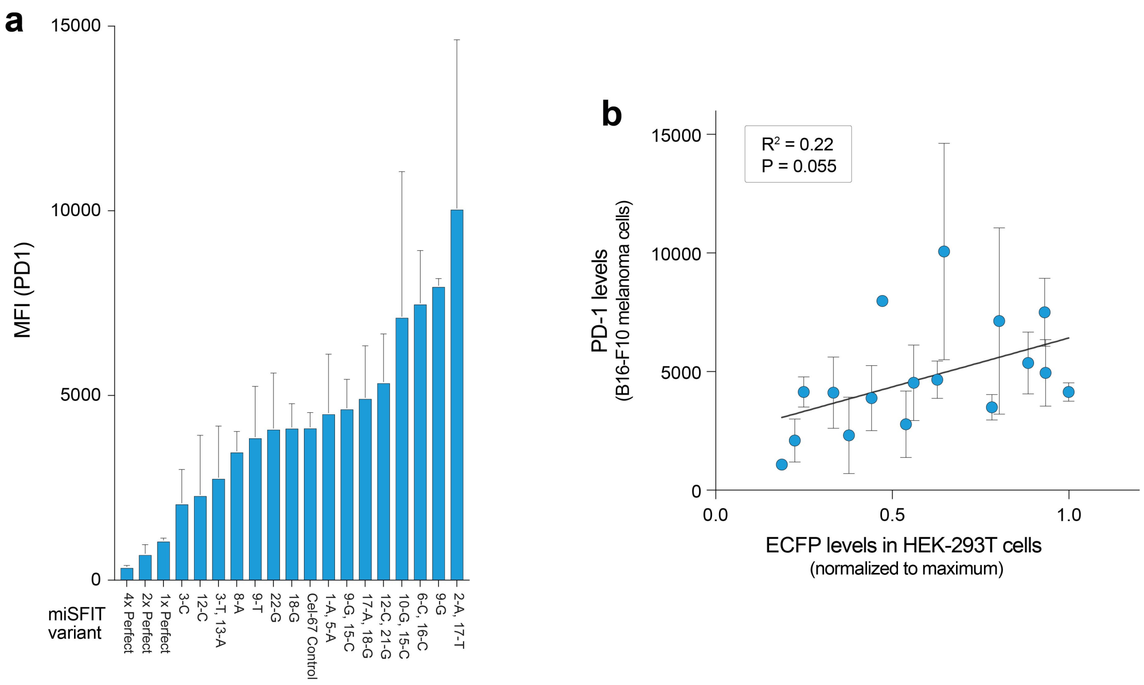

Supplementary Figure 9. miSFITs confer stepwise tuning of PD-1 in mouse B16-F10 cells. (a) Flow cytometry analysis of PD-1 expression in B16-F10 mouse melanoma cells transiently transfected with a panel of 19 miSFIT variants (n = 3 biological replicates, mean + s.d.). Analysed events were gated on NGFR, an internal transfection control. **(b)** Linear regression analysis comparing miSFIT expression levels for ECFP in HEK-293T cells versus PD-1 in B16-F10 cells. *P* value indicates that slope of a linear regression does not significantly differ from 0 (n = 17 variants, 3 biological replicates, mean +/- s.d.). Source data are provided as a Source Data file.

Reagent	Fluorophore	Supplier	Clone	Catalogue number
TruStain fcX (anti CD16/32)	None	BioLegend	93	101320
Live-dead	NIR	BioLegend		423105
CD4	BUV805	BD	GK1.5	564922
CD8	BV711	BioLegend	53-6.7	100747
TCRb	APC	BioLegend	H57-597	109212
CD45.1	PE	BioLegend	A20	110707
CD45.2	PerCP-Cy5.5	BioLegend	104	109828
CD69	BUV737	BD	H1.2F3	564684
CD25	BUV395	BD	Pc61	564022
CD44	AF594	BioLegend	IM7	103054
CD62L	FITC	BioLegend	MEL-14	104405
CTLA4	BV421	BioLegend	UC10-4B9	106311
PD1	PE-Cy7	BioLegend	29F.1A12	135216
Human PD1	PE	eBioscience (Invitrogen)	J105	12-2799-41
NGFR (CD271)	V450	BD	C40-1457	562123
Human PDL1	APC	BioLegend	29E.2A3	329708
Precision counting beads	None	BioLegend		424902

Supplementary Table 1. Flow cytometry reagents used in the study
