# Supplementary material for: Precise tuning of gene expression levels in mammalian cells
Source: Nat Commun. 2019 Feb 18;10:818. doi: 10.1038/s41467-019-08777-y (PMC6379387; doi:10.1038/s41467-019-08777-y)
Supplement: Supplementary file 2 — Description of Additional Supplementary Files [file 41467_2019_8777_MOESM2_ESM.docx]

**Title:** Supplementary Dataset 1

**Description:** Oligonucelotide sequences used in the study
