# Supplementary material for: Precise tuning of gene expression levels in mammalian cells
Source: Nat Commun. 2019 Feb 18;10:818. doi: 10.1038/s41467-019-08777-y (PMC6379387; doi:10.1038/s41467-019-08777-y)
Supplement: Supplementary file 5 — Reporting Summary [file 41467_2019_8777_MOESM5_ESM.pdf]

nature research

Corresponding author(s): *Double-blind peer review submissions: write DBPR and your manuscript number here instead of author names.*

Reporting Summary

Nature Research wishes to improve the reproducibility of the work that we publish. This form provides structure for consistency and transparency in reporting. For further information on Nature Research policies, see Authors & Referees and the Editorial Policy Checklist.

Statistical parameters

When statistical analyses are reported, confirm that the following items are present in the relevant location (e.g. figure legend, table legend, main text, or Methods section).

n/a	Confirmed	
☐	☒	The exact sample size (*n*) for each experimental group/condition, given as a discrete number and unit of measurement
☐	☒	An indication of whether measurements were taken from distinct samples or whether the same sample was measured repeatedly
☐	☒	The statistical test(s) used AND whether they are one- or two-sided<br>*Only common tests should be described solely by name; describe more complex techniques in the Methods section.*
☒	☐	A description of all covariates tested
☒	☐	A description of any assumptions or corrections, such as tests of normality and adjustment for multiple comparisons
☐	☒	A full description of the statistics including central tendency (e.g. means) or other basic estimates (e.g. regression coefficient) AND variation (e.g. standard deviation) or associated estimates of uncertainty (e.g. confidence intervals)
☐	☒	For null hypothesis testing, the test statistic (e.g. *F*, *t*, *r*) with confidence intervals, effect sizes, degrees of freedom and *P* value noted<br>*Give P values as exact values whenever suitable.*
☒	☐	For Bayesian analysis, information on the choice of priors and Markov chain Monte Carlo settings
☒	☐	For hierarchical and complex designs, identification of the appropriate level for tests and full reporting of outcomes
☐	☒	Estimates of effect sizes (e.g. Cohen's *d*, Pearson's *r*), indicating how they were calculated
☐	☒	Clearly defined error bars<br>*State explicitly what error bars represent (e.g. SD, SE, CI)*

Our web collection on statistics for biologists may be useful.

Software and code

Policy information about availability of computer code

Data collection	Flow cytometry data were collected with BD FACSDIVA
Data analysis	Graphpad Prism V7, FlowJo V10.3, R V3.4.1, Biostrings V2.44.2 and custom R functions that are available upon request

For manuscripts utilizing custom algorithms or software that are central to the research but not yet described in published literature, software must be made available to editors/reviewers upon request. We strongly encourage code deposition in a community repository (e.g. GitHub). See the Nature Research guidelines for submitting code & software for further information.

Data

Policy information about availability of data

All manuscripts must include a data availability statement. This statement should provide the following information, where applicable:

- Accession codes, unique identifiers, or web links for publicly available datasets
- A list of figures that have associated raw data
- A description of any restrictions on data availability

Raw HTS data (Fastq files) have been deposited into the Sequence Read Archive (SRA) at the European Nucleotide Archive (ENA). SRA accession: PRJNA516224. All other raw data that is not found in the supplementary information is available from the corresponding author upon reasonable request.

Field-specific reporting

Please select the best fit for your research. If you are not sure, read the appropriate sections before making your selection.

☒ Life sciences ☐ Behavioural & social sciences ☐ Ecological, evolutionary & environmental sciences

For a reference copy of the document with all sections, see nature.com/authors/policies/ReportingSummary-flat.pdf

Life sciences study design

All studies must disclose on these points even when the disclosure is negative.

Sample size	A sample size of n=3 to n=5 were chosen for cell culture experiments to enable robust statistical analysis. In vitro transfection and co-cultures showed low variability. For in vivo experiments we aimed for n=5 to n=6 animals per group to balance statistical power with animal usage reduction.
Data exclusions	Mice that did not have detectable tumours by day 17 were excluded from the study. This criteria was per-determined.
Replication	In addition to the replicates reported in this study, T-cell activation and killing experiments were repeated on an independent day with reproducible results. A small scale in vivo tumour growth replication experiment was also undertaken (n=3 or n=4 mice per condition) that reproduced the reported findings.
Randomization	Mice were allocated to experimental groups arbitrarily. See below for blinding.
Blinding	The experimenter who measured in vivo tumour growth was blinded by a co-author to the experimental condition of each mouse. An ear notch system was used to record the data.

Reporting for specific materials, systems and methods

Materials & experimental systems

n/a	Involved in the study	
☒	☐	Unique biological materials
☐	☒	Antibodies
☐	☒	Eukaryotic cell lines
☒	☐	Palaeontology
☐	☒	Animals and other organisms
☒	☐	Human research participants

Methods

n/a	Involved in the study	
☒	☐	ChIP-seq
☐	☒	Flow cytometry
☒	☐	MRI-based neuroimaging

Antibodies

Antibodies used	For ease of access, this information is all provided in supplementary table 2
Validation	The antibodies used in the study have been validated by the supplier for use in flow cytometry

Eukaryotic cell lines

Policy information about cell lines

Cell line source(s)	HEK293T cells and B16F10 cells were purchased from ATCC. Jurkat 1.G4 cells were a gift from Simon Davis
Authentication	We did not independently authenticate these cell lines
Mycoplasma contamination	All cell lines tested negative for mycoplasma contamination in a PCR-based assay.
Commonly misidentified lines (See ICLAC register)	None

Animals and other organisms

Policy information about studies involving animals; ARRIVE guidelines recommended for reporting animal research

Laboratory animals	We used female C57BL/6 mice aged 6 to 10 at the outset of experiments. Female OT-1(C57BL/6 background) mice aged 6 to 12 weeks were used as a source of primary splenocytes
Wild animals	This study did not use wild animals
Field-collected samples	We did not collect field samples

Flow Cytometry

Plots

Confirm that:

☒ The axis labels state the marker and fluorochrome used (e.g. CD4-FITC).

☒ The axis scales are clearly visible. Include numbers along axes only for bottom left plot of group (a 'group' is an analysis of identical markers).

☒ All plots are contour plots with outliers or pseudocolor plots.

☒ A numerical value for number of cells or percentage (with statistics) is provided.

Methodology

Sample preparation	For details see the materials and methods section. Briefly, adherent cells were harvested with trypsin, cells were washed, blocked as necessary and stained on ice. Cells were washed again prior to analysis.
Instrument	All flow-cytometry experiments were performed on the BD LSR Fortessa Analyzer or the FACSymphony (BD Biosciences)
Software	Data were analysed using FlowJo (Version 10.3.0)
Cell population abundance	Post sort purity was determined by subsequent flow cytometry.
Gating strategy	Population gating strategies are shown in supplementary figures 3,4,5 and 6. In general cells were gated on FSC-A vs SSC-A and then FSC-A vs FSC-H. Subsequent gates were drawn based on un-transfected, un-transduced or un-stained controls.

☒ Tick this box to confirm that a figure exemplifying the gating strategy is provided in the Supplementary Information.
